# The Emerging Roles of Gamma–Delta T Cells in Tissue Inflammation in Experimental Autoimmune Encephalomyelitis

**DOI:** 10.3389/fimmu.2016.00014

**Published:** 2016-01-29

**Authors:** Sakshi Malik, Muzamil Yaqub Want, Amit Awasthi

**Affiliations:** ^1^Translational Health Science and Technology Institute, Faridabad, India

**Keywords:** gamma–delta T cells, Th17 cells, cytokines, inflammation, autoimmunity

## Abstract

γδ (gamma–delta) T cells, a small population of unconventional T cells, have been found in central nervous system lesions of multiple sclerosis (MS) patients, but their function in disease activity is not clearly understood. Previous studies in experimental autoimmune encephalomyelitis (EAE) were inconsistent in identifying their specific roles in suppressing or promoting disease pathogenesis. Emerging advancements in the biology of γδ T cells especially in the context of their being the major initial producers of IL-17, suggested their crucial role in pathogenesis of EAE. In addition, γδ T cells express high levels of IL-23R and IL-1R, which further enhance their effector functions in the pathogenesis of EAE. Nonetheless, activated heterogeneous γδ T cells display functional dichotomy, which is crucial in determining the outcomes of tissue inflammation in EAE. In this review, we discussed recent advances in understanding the biology of γδ T cells in tissue inflammation as well as their roles in suppressing or promoting the development of EAE.

## Introduction

γδ (gamma–delta) T cells comprise a small fraction (~1–5%) of the total blood lymphocytes of mice and humans and are more commonly localized in mucosal tissue and skin where they constitute a major population (up to 50%) of lymphocytes (1). The identification of an unusually rearranged γ chain of the T cell receptor (TCR) gene led to the discovery of γδ T cells (2, 3). After the identification of γδ T cells as a new subset of T cells, it became clear that these cell types, unlike their αβ (alpha-beta) T cell counterparts, possess features of both innate and adaptive immune cells (4, 5). Moreover, γδ T cells have also been recognized as non-conventional innate-like cells as they share several features of innate immune cells, such as surface expression of Toll-like receptors (TLRs) (6). In addition, γδ T cells acquire preactivated phenotypes of effector and memory T cells during their early development (6).

The antigen recognition, activation, and effector functions of γδ T cells are different than those of their αβ T cell counterparts. Unlike αβ^+^ T cells, γδ T cells can be activated with or without their cognate TCR ligands and appear to induce an early burst of inflammatory cytokine that initiates effective and progressive αβ T cell responses in tissue inflammation during experimental autoimmune encephalomyelitis (EAE) (7–10). These unusual unique features of γδ T cells make them an early effector T cells during an immune response in inflamed tissue.

αβ^+^ CD4^+^ T cells are crucial for inducing tissue inflammation in EAE. It has been convincingly elucidated that IL-17-producing Th17 cells are the major driver in inducing pathogenesis of EAE. Ablation of Th17 cells or absence of IL-17 significantly reduces the severity of inflammation in EAE (11). Similarly, the absence of Th17 cell-associated genes, such as *Rorc*, a master transcription factor for Th17 cells development, and IL-23R also attenuate inflammation in EAE (12, 13). Interestingly, γδ T cells express higher level of IL-23R on their surface, which raised an interesting possibility that IL-23-responsive γδ T cells may contribute to the severity of tissue inflammation in EAE (8). Furthermore, GWAS studies suggested a genetic association of IL-23R with MS (14).

Although the *bona fide* antigens were identified for γδ T cells, still not much is known about their antigenic repertoire and restrictions (15). In addition to their antigens, γδ T cells can be activated by TLRs to induce various inflammatory cytokines, such as IFN-γ, IL-4, IL-17, IL-21, and IL-22 (6, 16).

Unlike αβ^+^ T cells, antigen recognition by the TCR of γδ T cells does not require antigen processing and presentation by MHC molecules (17, 18). Moreover, deficiencies of MHC class II and β2 microglobulin do not affect the development of γδ T cells and their repertoire remain intact, which suggest that the generation of γδ T cells is apparently independent of both class I and II molecules (19, 20). Interestingly, non-classical MHC class Ib molecules T10 and T22 are described as the natural ligands for murine γδ T cells (21, 22). Similarly, human class I-like molecules MICA and MICB were also suggested as natural antigens for human γδ T cells (21, 23–25). Interestingly, alterations in the expression of these ligands are induced by infection or tissue inflammation or stress, which can provide early danger-signal to initiate the activation of γδ T cells even in the absence of αβ^+^ T cells activation (15, 16).

The functions of γδ T cells in different pathophysiological conditions are driven by their tissue-specific distributions and tropism. At steady state, γδ T cells are predominantly localized in epithelial surfaces of liver, skin, and mucosal surfaces of digestive, respiratory, and reproductive organs (15, 16). Moreover, the distribution of γδ T cells to the above mentioned epithelial and mucosal surfaces is often driven by their specific expression of invariant or closely related γδ TCRs; for example, Vγ6Vδ1 TCR-expressing γδ T cells mostly accumulate in the lung, peritoneum, and reproductive organs, while Vγ5Vδ1-bearing γδ T cells predominantly reside in the epithelial surface of the skin (16). In addition to their tissue localization, cellular distribution, pathophysiological conditions, and inflammatory signals also determine the activation and phenotypic plasticity of γδ T cells.

Upon activation, γδ T cells can produce the effector cytokines of Th1, Th2, and Th17 cells, such as IFN-γ, IL-4, and IL-17, respectively, therefore contribute to specific effector function in Th1, Th2, and Th17 cell-associated tissue inflammation (26). Interestingly, IL-23 stimulation of γδ T cells rapidly induces IL-17 production (6, 13, 27) to initiate tissue inflammation and enhance CD4^+^ αβ Th17 cells responses during EAE (7). It is apparent that γδ T cells play critical role in the induction and pathogenesis of EAE (15). Nonetheless, the regulatory role of γδ T cells is also suggested in EAE.

## Subsets of γδ T Cells and Their Functions in EAE

The functions of γδ T cells are not only critically required for elimination of intra- and extracellular pathogens and tissue surveillance in cancer but are also associated with multiple organ-specific autoimmunity, such as type 1 diabetes, arthritis, inflammatory bowel disease (IBD), and MS (16).

There are multiple subtypes of γδ T cells that are involved in the pathogenesis of EAE and can be identified based on the usage of their variable regions for both γ and δ genes (28, 29). Unlike the mucosal surfaces and the skin, which usually harbor higher frequency of γδ T cells, a smaller frequency of γδ T cells can be found within the central nervous system (CNS) in steady state of untreated naive mice (30, 31). Although the role of γδ T cells in the CNS at steady state is not precisely understood, it might be possible that their presence within the CNS could be required for carrying out immune surveillance function. Nonetheless, the frequency of γδ T cells profoundly increases within the CNS in EAE; and moreover, their distribution within the CNS can be classified based on their TCR usage during different phases of EAE (28). At the initial phase of EAE, CNS-infiltrating γδ T cells show a limited repertoire, including Vδ1, Vδ4, Vδ5, Vγ1–3, and Vγ6, while almost all the Vγ and Vδ transcripts can be found in the brain at the chronic or later phase of the disease (28). Although lymph nodes of EAE mice contained most of the Vγ transcripts during all phases of disease, a limited repertoire of γδ T cells was also observed within the CNS at the initial phase of the disease. Though Vγ6 (also known as DV7s6) expressing γδ T cells are predominantly located in mucosa, but they can also be found within the CNS at the initial phase of EAE. However, the precise antigen specificity of CNS-localized Vγ6 γδ T cells is not clearly understood in EAE. Since γδ T cells do not appear to recognize myelin basic protein (MBP) as antigen; therefore, Vγ6 T cells might be recruited to the CNS in EAE in response to the heat shock protein (HSP), which is expressed on stressed autologous cells (32). In addition, another possibility is that Vγ6 γδ T cells could recognize self-antigens that mimic bacterial peptide in the CNS during inflammation as this subset of γδ T cells is known to recognize microbial antigens (bacterial peptide) at mucosal surfaces (15, 33). Furthermore, γδ T cells are suggested to be functionally dichotomous on the basis of their TCR usage in EAE; Vγ1 subset preferentially regulates while Vγ4 subset further enhances tissue inflammation in EAE (34). Further analysis revealed that the Vγ1 subset is predominantly prevalent in spleen in all phases of EAE, and in fact, about 35–50% of total splenic γδ T cells are found to be Vγ1 in EAE (34). However, a small percentage of Vγ1 γδ T cells are also found in the CNS during EAE. Emerging literature suggested that Vγ1 γδ T cells act as regulatory cells and were shown to suppress tissue inflammation during the acute phase of EAE by enhancing the functions of Foxp3^+^ regulatory T (Treg) cells. Moreover, it is proposed that Vγ1 subset of γδ T cells highly express CCL4, which can bind to CCR5 on Treg cells and promote their suppressive functions in EAE (34). Consistent with their regulatory role in EAE, CNS-sorted Vγ1 γδ T cells from EAE mice do not express high amounts of IL-17A, IL-17F, IL-23R, and GM-CSF, which further reinforce their regulatory function in EAE (34). Thus, it is suggested that Vγ1 subset might shift the balance away from Th17 cells while promoting the proliferation and suppressive functions of Treg cells during EAE.

Yet, another subset of γδ T cells, Vγ4 predominates in the CNS during EAE. These cells typically responds to self-antigens by producing pro inflammatory cytokine, such as IL-17, which in turn can directly act on stromal cells and induce migration of lymphocytes across blood brain barrier in EAE (8, 27). Interestingly, the IL-17-producing Vγ4 γδ T cells also expressed other Th17 cell-associated molecules, such as Rorc, IL-22, IL-1R, and IL-1β (6, 34), which further suggested to contribute to inflammation and exacerbation of EAE (34). In addition to EAE, IL-17-producing Vγ4 γδ T cells are shown to promote collagen-induced arthritis (CIA), as antibody-mediated depletion of Vγ4 γδ T cells resulted in attenuated tissue inflammation in CIA (35). It is proposed that adjuvant rather antigen expands IL-17-producing Vγ4 γδ T cells in CIA.

The ability of γδ T cells to produce IL-17 innately in response to IL-23 in EAE could be attributed to Vγ4 subset of γδ T cells as they highly express IL-23R on their surface (6, 7, 27). Nonetheless, it is not clearly understood whether natural ligand or antigen of Vγ4 γδ T cells can induce strong IL-17 response in EAE. In addition to Vγ4 γδ T cells, Vγ6 γδ T cells, which primarily resides under the skin also express IL-23R on their surface, and therefore might be contributing to IL-17-mediated inflammation in the CNS of EAE mice (27).

Interestingly, in addition to IL-23R and Vγ4, the differential expression of CD27 can also identify γδT17 cells (IL-17-producing γδ T cells). CD27^+^ γδ T cells produce IFN-γ while CD27^−^ γδ T cells secrete IL-17 suggested that the surface expression CD27 can differentially mark IL-17- and IFN-γ-producing γδ T cells (36).

Furthermore, structural and functional heterogeneity of γδ T cells in EAE can be further contributed by different mice strain. Olive et al. have reported amplification of Vγ5 transcript in C57Bl/6 mice during EAE while this transcript was not detected in the CNS of SJL/J mice, suggesting that the infiltrating population of γδ T cells in CNS during disease can be varied on the basis of mouse strains (28).

## Th17 Cells Differentiation and IL-17-Producing γδ T Cells in EAE

After the identification of Th17 cells as a separate lineage of helper T (Th) cells, it became clear that they, together, with Th1 cells, play a crucial role in EAE (37, 38). Before the identification of Th17 cells, IFN-γ-producing Th1 cells were thought to be the primary effector cell type involved in the disease induction of EAE, which has puzzled immunologist for a very long time as both IFN-γ- and IFN-γR-deficient animals had exacerbated tissue inflammation in EAE (39). In addition, the deficiencies of IL-12p35 (IL-12) and IL-12Rβ2 (IL-12 receptor), which are critically required for the development of Th1 cells, also enhanced the development of EAE (37). Taken together, it is clearly suggested that Th1 cells are not the primary effector T cell subsets involved in development of EAE. In fact, Th1 cell-associated molecules, such as IFN-γ, IL-12, and IL-12R, negatively regulate disease and tissue inflammation in EAE (11). Nonetheless, Th1 cells also critical for the development of EAE, as Th1 cells were found in the CNS in active EAE. In fact, a sizable population of IFN-γ and IL-17 double positive CD4^+^ T cells was found within the CNS at the peak of EAE (40).

Seminal studies demonstrated that TGF-β1 and IL-6 are required for the differentiation of Th17 cells (41–43). IL-6 strongly induces IL-21 in Th17 cells, which creates feed forward loop to further amplify the generation of Th17 cells (44–46). The role of Th17 cells and IL-17 was further demonstrated by using IL-17-deficient mice, as *Il-17*^−/−^ animals develop attenuated EAE with delayed onset. Moreover, the adoptive transfer of *Il-17*^−/−^ CD4^+^ T cells is inefficient in transferring EAE, suggesting that IL-17 is crucial for tissue inflammation and disease pathogenesis (47).

Similar to Th17 cells, IL-6 and TGF-β are also crucial for the generation of γδT17 cells (8, 48). *Tgfb*^−/−^ and *Smad3*^−/−^ mice harbor reduced precursor frequency of γδT17 cells in thymus (48). On the other hand, *Il6*^−/−^ mice have shown reduced frequency of peripheral γδT17 cells (8). Taken together, similar to Th17 cells differentiation, TGF-β and IL-6 are crucial for the generation of γδT17 cells.

Importantly, the precise role of γδ T cells was demonstrated in EAE using *Tcrd*^−/−^ mice (15, 49). Mice lacking TCR delta chain gene develop less severe EAE with reduced infiltration of αβ^+^ T cells in their CNS (49). Similarly, depletion of γδ T cells by anti-GL3 antibody before the onset or at chronic phase of EAE reduces the severity and clinical signs of EAE (50). Moreover, antibody-mediated depletion of γδ T cells regulates the influx of proinflammatory cytokines, such as IL-1, IL-6, TNF-α, lymphotoxin, and IFN-γ, further suggesting an essential role of γδ T cells in contributing to the pathogenesis of EAE (50). Furthermore, it is demonstrated that the depletion of γδ T cells from MBP-reactive lymph node cells transferred attenuated EAE with reduced T cells proliferation and IL-12 secretion (51). Moreover, replenishing γδ T cells population not only enhanced the severity of EAE but also restored the IL-12 production and T cells proliferation (51).

In addition, a detailed systematic analysis of γδ T cells was performed to understand their distribution in different phases of EAE (52). Interestingly, an increased frequency of γδ T cells (up to 12% of total CD3^+^ T cells) was found in the CNS during the acute phase while the percentage of γδ T cells decreased (from 12 to 5% of total CD3^+^ T cells) during the recovery phase of EAE (52). Since the frequency of myelin-specific Foxp3^+^ Treg cells increases during recovery phase of EAE, it is possible that the increased number of Foxp3^+^ Tregs contributes in controlling the expansion of γδ T cells population during recovery phase of EAE (8, 53). Interestingly, the contraction of γδ T cells population was restricted only to the CNS, as their percentages in spleen remained low (~2% of total CD3^+^ T cells) during all phases of EAE. This implies that γδ T cells selectively accumulate in the target tissue during tissue inflammation to enhance severity of inflammation in EAE (52).

Although αβ^+^ CD4^+^ T cells are suggested to be the primary source of IL-17 in infection and autoimmune inflammation, γδT cells can be a potent source of IL-17, and in some cases, even more dominant than Th17 cells (6, 33, 54). In fact, in the model of Fas-ligand-induced inflammation in which injecting FasL-expressing tumor cells into peritoneum of mice induces enhanced production of IL-17 from non-conventional T cells (55). Interestingly, the majority of these IL-17-producing cells were γδ T cells as compared to αβ Th17 cells in this particular model (55). Similarly, γδ T cells isolated from *Mycobacterium*-infected lung and spleen produce massive amounts of IL-17 as compared to αβ Th17 cells (56). Furthermore, in other model of infection, such as *Escherichia coli*, *Bacillus subtilis*, and experimental sepsis, γδ T cells, rather than αβ^+^ Th17 cells, are the primary source of IL-17 (33, 57). Hence, in certain conditions, γδ T cells appear to have an inherent ability to rapidly produce substantial amounts of IL-17 without being primed.

Although, initial studies identified that IL-17-producing γδ T cells are essential for clearing infections, the role of γδ T cells are also suggested for inducing autoimmune inflammation and propagation of autoimmune diseases, including EAE (15).

In addition to Th17 cells, Th1 cells were also implicated in the development of EAE (58). In fact, many studies suggested that myelin-specific Th1 cells adoptively transfer EAE (58). Interestingly, the initiation of EAE development by adoptively transferred myelin-specific Th1 cells resulted in recruitment of IL-17-producing host cells (IL-17hc) to the CNS (59). Further cellular characterization revealed that γδ T cells comprising almost 60% of the total IL-17hc (59). Moreover, in the absence of IL-17hc, myelin-specific Th1 cells transferred less severe EAE, suggesting the requirement of host production of IL-17, largely by γδ T cells, in the development of EAE (59).

## Proinflammatory Cytokines that Induce IL-17 from γδ T Cells in Inflammation in CNS During EAE

Progression and development of tissue inflammation in EAE are primarily mediated by infiltrating mononuclear cells, which produce proinflammatory cytokines. Among other CNS-infiltrating cells, γδ T cells predominantly and rapidly produce proinflammatory cytokines to further enhance tissue inflammation in EAE. Like conventional αβ T cells, γδ T cells also expand in secondary lymphoid organs upon immunization with MOG/CFA. Once migrated to the CNS in β2 integrin-independent manner, these γδ T cells further expand and accumulate shortly before the peak of EAE and produce IFN-γ, TNF-α, and IL-17 to further enhance disease progression (60, 61).

**Figure 1:**
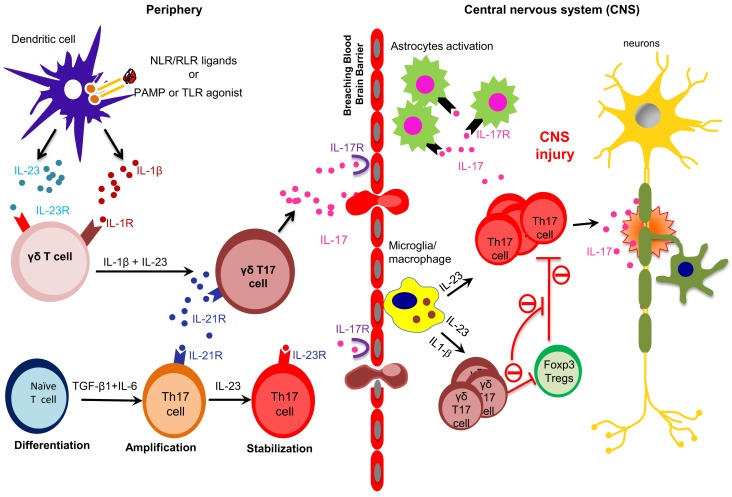
**Peripherally primed γδ T cells execute their effector functions in the CNS in EAE**. TLRs and NLRs activated dendritic cells (DCs) and macrophages produce proinflammatory cytokines, such as IL-6, IL-23 and IL-1β. IL-23 and IL-1β are sensed by IL-23R-expressing γδ T cells, which in turn produce early burst of IL-17 during early phase of EAE. On the other hand, IL-6 together with TGF-β induce the differentiation of Th17 cells. γδT17 cells produce IL-21, which further amplify their own generation and also amplify the generation of Th17 cells. Differentiated γδT17 and αβ Th17 cells breach the blood brain barrier to execute their effector functions within the CNS during EAE. Activated microglia/macrophages produce IL-23 within the CNS to promote the generation of γδT17 and Th17 cells. Inflammatory γδT17 cells promote CNS injury in EAE by enhancing the effector functions of Th17 cells and restraining the suppressive functions of Tregs cells.

Unlike αβ Th17 cells, which require primary (TCR), secondary (costimulation) and cytokine signals (TGF-β1 + IL-6) to produce IL-17, γδ T cells can produce IL-17 with cytokine signals (IL-23 and IL-1β) alone in the absence of primary and secondary signals (7). This peculiar feature of γδ T cells make them superior IL-17 producers by capturing the initial burst of proinflammatory cytokines produced by dendritic cells (DCs) and macrophages in response to TLR and NLR activation in EAE. The ability of γδ T cells to generate an initial burst of IL-17 in the absence of activation of αβ T cells is critical for initiating CNS inflammation, as *Tcrd*^−/−^ mice develop less severe EAE with reduced production of IL-17 (7, 49). Moreover, αβ T cells from *Tcrd*^−/−^ mice produce lower amounts of IL-17 as compared to αβ T cells from wild-type mice (8), which clearly suggested that the presence of γδ T cells is essentially required for optimal production of IL-17 by αβ T cells. Interestingly, *Il1r*^−/−^ mice are substantially more resistant to EAE development (62); however, reconstituting IL-1R-sufficient γδ T cells into *Il1r*^−/−^ mice prior to MOG immunization enhances progression of EAE, suggesting that IL-1β–IL-1R interaction on γδ T cells is essential for promoting tissue inflammation in EAE (7). Furthermore, stimulation of γδ T cells with IL-1β together with IL-23 synergistically enhanced IL-17 production in the absence of TCR stimulation (7). In addition to IL-17, other Th17 cell-associated cytokines, such as IL-17F, IL-21, and IL-22, were also produced by γδ T cells upon their activation with IL-1β and IL-23 (Figure 1). Consistently, culture supernatant of IL-1β− and IL-23-stimulated γδ T cells further enhanced IL-17 production from αβ^+^ CD4^+^ T cells (7, 8). Neutralization of IL-21 and IL-17 reduced IL-17 induction from αβ^+^ CD4^+^ T cells induced by culture supernatant of IL-1β- and IL-23-stimulated γδ T cells (7, 8). In fact, it is suggested that the combination of IL-1β- and IL-23-stimulated γδ T cells provides early burst of IL-21, which not only enhances production of IL-17 by the γδ T cells but it can also amplify the generation of Th17 cells (8, 11, 45) (Figure 1).

In addition to IL-21, another common γ chain family cytokine, IL-2 also play a role in generation of γδT17 cells. IL-2, which is known to suppress Th17 cells (63), promotes γδT17 cells generation, as *Il2*^−/−^ and *Cd25*^−/−^ mice selectively reduced the frequency of γδT17 cells (64). Interestingly, the new subset of IL-15-producing γδ T cells (γδT15) was recently identified in EAE (65). γδT15 cells suggested to enhance tissue inflammation in EAE by enhancing the functions of CD44^hi^ memory T and Th17 cells (65). However, whether these γδT15 cells express other inflammatory cytokines, such as IL-17 and GM-CSF, are not clear. In summary, various cytokines signals are required for the generation of γδT17 cells; and interestingly, some of these cytokines can directly activate γδ T cells without the requirement of TCR activation. Taken together, the initial burst of proinflammatory cytokines produce by γδ T cells is crucial for induction of EAE.

## IL-18 Promotes IL-17 Induction from γδ T Cells in EAE

**Figure 2:**
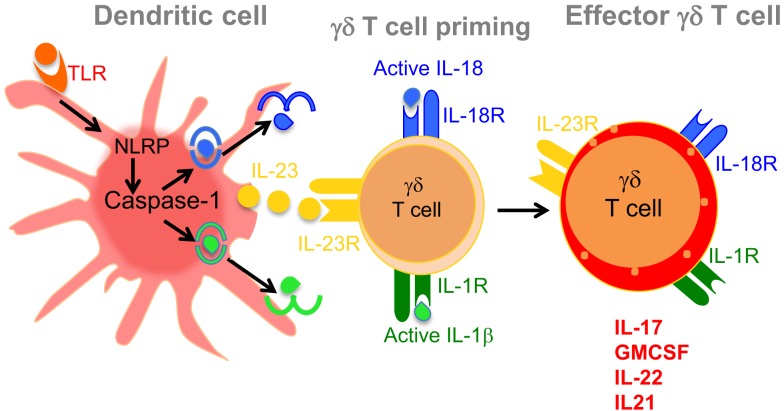
**Dendritic cell-derived IL-23, IL-1β, and IL-18 mediates induction of effector γδT17 cells**. Ligation of TLRs on the surface of DCs induces caspase-1 activation in inflammasome-dependent manner. Activated caspase-1 cleaves pro IL-1β and IL-18 into their active forms as shown in the figure. Activated DCs also produce IL-23, which together with IL-1β or IL-18 promote the induction of proinflammatory cytokines, such as IL-17, GM-CSF, IL-21, and IL-22. These effector γδ T cells initiate disease induction and help αβ^+^ CD4^+^ T cells to induce EAE.

IL-18, an IL-1 family cytokine, also known as IFN-γ-inducing factor. It has been shown that IL-18 further enhances the development of IL-12-induced Th1 cells. Moreover, Th1 cells sensitized with IL-18 enhance their disease promoting effector functions in EAE by activating IFN-γ-producing NK cells (66). The function of IL-18 in EAE was described using IL-18R1-deficient animals. *Il18r1*^−/−^ mice were completely resistant to development of EAE, suggesting the role of IL-18R in inducing encephalitogenic T cells in disease (67). Moreover, the engagement of IL-18Rα on antigen-presenting cells is essential for generation of pathogenic Th17 cells during EAE (67). In fact, caspase-1-processed cytokines IL-1β and IL-18 predominantly promote innate production of IL-17 from γδ T cells in EAE (9). Immunization with CFA, which contains heat-killed cell wall of *Mycobacterium tuberculosis*, activates caspase-1 via NLRP3 inflammasome to induce active forms of IL-1β and IL-18 from DCs. Inhibition of caspase-1 by its specific inhibitor suppresses EAE development and IL-17 production from γδ T cells (9). Similar to IL-23R, γδ T cells also express IL-18R constitutively on their surface even in the steady state (Figure 2). On the contrary, the expression of IL-18R on CD4^+^ T cells is induced in inflammatory conditions during EAE, suggesting that γδ T cells, and not CD4^+^ T cells, respond first to the IL-18 in order to induce IL-17 production. It has been shown that the combination of IL-18 together with IL-23 rapidly induced innate production of IL-17 from γδ T cells in the absence of TCR stimulation (Figure 2). This initial burst of IL-17 from γδ T cells may be required for initiation of EAE and the development of pathogenic Th17 cells. It is, however, unclear whether coexpression of IL-23R and IL-18R on γδ T cells make them more pathogenic in initiating EAE.

## GM-CSF-Producing γδ T in Tissue Inflammation During EAE Development

In addition to IL-17 and IFN-γ, GM-CSF is also essentially required for the development of EAE. GM-CSF-deficient mice are resistant to the development of EAE with reduced infiltration of effector T cells into the CNS (68). Rostami et al. reported that the neutralization of GM-CSF-attenuated tissue inflammation in EAE (69). Taken together, it is clearly suggested that GM-CSF is required for the induction of encephalitogenic T cells in EAE. In fact, both Th1 and Th17 cells were shown to produce GM-CSF, which can further enhance the encephalitogenicity of these effector T cells in mediating the development of EAE. Moreover, it is proposed that GM-CSF is critical for the induction of pathogenic Th17 cells in EAE. Although both IL-12 and IL-23 can induce the production of GM-CSF by the effector T cells, it is clearly demonstrated that IL-23, but not IL-12, signaling is critically required for GM-SCF production in EAE (70–72). Similarly, exposure of IL-23 enhances the pathogenic functions of Th17 cells mediated by GM-CSF in EAE. In addition to CD4^+^ T cells, macrophages, and NK cells, γδ T cells produce high amounts of GM-CSF, which contributes to neuroinflammation of CNS in EAE (68). In fact, γδ T cells are the major innate source of GM-CSF in the CNS during EAE development (10). Combination of IL-23 together with IL-1β promotes GM-CSF production from γδ T cells in the absence of TCR stimulation (10). Moreover, the production of GM-CSF induced by IL-23 and IL-1β was compromised in *Il1r*^−/−^ γδ T cells. In fact, production of GM-CSF by CNS-infiltrating γδ T cells is abolished in *Il1r*^−/−^ mice, suggesting that IL-1 signaling is crucial for generation of GM-SCF-producing γδ T cells in EAE (Figure 2) (10). Caspase-1, which is required for active IL-1β production, is also critical in inducing GM-CSF from γδ T cells, as *caspase1*^−/−^ γδ T cells are defective in GM-CSF production. In fact, *caspase1*^−/−^ and *Il1b*^−/−^ mice share a similar EAE phenotype, suggesting a specific role of caspase-1 and downstream IL-1β in regulating the induction of GM-CSF during EAE (10). It is suggested that GM-CSF contribute to the development of EAE by enhancing the functions of CNS-resident myeloid cells, including microglial cells (70). Although IL-1 signaling is required for the generation of GM-CSF-producing γδ T cells within the CNS during EAE, it is not identified which subtype of γδ T cells predominantly produce GM-CSF during disease. Moreover, IL-1- and IL-23-mediated inductions of GM-CSF in γδ T cells are dependent on MyD88 signaling, as γδ T cells from MyD88-deficient mice severely reduced GM-CSF production (10). Since MyD88 is a major downstream signaling component of TLR signaling pathway, it might be possible that ligation of TLRs on γδ T cells can also induce IL-17 production (57). To precisely understand the role TLRs in generating γδT17 cells, Dong et al. used IL-17-RFP⋅KI mice to understand the cellular source of IL-17 in EAE in response to TLR4 ligation. Using a faithful IL-17 reporter system, Dong et al. clearly demonstrated that the expression of TLR4 is high on IL-17^+^ as compared to IL-17-γδ T cells (31). In addition to IL-17 expression, IL-23-stimulation strongly enhanced the expression of TLR4 on γδ T cells. Moreover, the combination of IL-23 together with LPS further enhanced the secretion of IL-17 from γδ T cells (31). In addition to induced IL-17 production, TLR4 signaling also enhanced the survival of γδ T cells, which can further contribute in enhancing tissue inflammation in EAE. Taken together, TLRs especially TLR4 plays an essential role in inducing the development of IL-17-producing γδ T cells and their survival.

## γδ T Cells Make αβ^+^ CD4^+^ T Cells Refractory to Treg Suppression in EAE

Regulatory T cells are critical for maintaining immune homeostasis of the host as loss of these cells either by naturally occurring mutation or cellular ablation leads to overwhelming activation of effector T cell-mediated multiple organ failure of the host (73–75). The critical functions of Treg cells were described in various models of autoimmune diseases, including EAE (53, 76). Using MOG tetramer and Foxp3-GFP⋅KI mice, it has been demonstrated that myelin–antigen-specific Treg cells are primed and expanded during the priming phase of EAE (53). Similar to effector T cells, these myelin-specific Treg cells can effectively migrate to the CNS (53). Although their frequency within the CNS is lower during the peak of EAE, strikingly, the population of Treg cells outnumber the population of effector T cells within the CNS at recovery phase of EAE (53). These CNS-accumulated Treg cells produce both IL-10 and TGF-β, which help in resolving the inflammation at the recovery phase of EAE. Interestingly, both Tr1 and Treg cells were shown to produce IL-10 in the CNS during the recovery phase of EAE (53, 77). In spite of their presence in the CNS at the peak of EAE, Treg cells failed to suppress proliferation and effector functions of CNS-accumulated effector T cells (53). Interestingly, cytokine analysis of CNS-accumulated effector CD4^+^ T cells revealed a strikingly higher production of proinflammatory cytokines, such as IL-6, TNF-α, and IL-21, which can be accounted for the failure of suppressive functions of Treg cells in EAE (53, 78). Interestingly, the higher frequency of γδ T cells together with αβ effector T cells found to be accumulated within the CNS (7, 8, 53, 76). In general, γδ T cells have high expression of IL-23R, in fact, all the γδ T cells present in the CNS at the peak of EAE exclusively expressed IL-23R (8, 13). Moreover, the frequency of IL-23R^+^ γδ T cells contracts while the frequency of Tregs cells increases during the recovery phase of EAE (8, 53). This raised an interesting possibility that the presence of γδ T cells within the CNS might promote the functions of inflammatory αβ^+^ T cells while hampering the suppressive functions of Treg cells in EAE (Figure 1). In fact, *Tcrd*^−/−^ mice mount-attenuated effector αβ T cells response in EAE, supporting the fact that the presence of γδ T cells are essential for effective CD4^+^ T cells effector functions in EAE (49). Interestingly, Korn et al. suggested a mechanism by which γδ T cells enhanced the effector functions of CD4^+^ T cells during inflammation (8). Activation of γδ T cells with IL-23 produced soluble factors, which make αβ^+^ T cells refractory to Treg cell-mediated suppression, as cellular supernatant of IL-23-activated γδ T cells inhibited the suppressive functions of Treg cell (7, 8). It has been demonstrated that Treg cells can lose their suppressive functions in the presence of inflammatory environment. In fact, IL-6 makes αβ effector T cells refractory to the suppressive activity of Tregs cells (8, 53, 78). In addition, IL-6 has also been shown to inhibit TGF-β-induced *de novo* conversion of conventional T cells into Treg cells (41, 42). Similarly, Kuchroo et al. has demonstrated that IL-21, in addition to IL-6, can also suppress TGF-β-mediated *de novo* conversion of conventional T cells into Treg cells. Interestingly, IL-23R-stimulated γδ T cells not only block the conversion of conventional T cells into Treg cells but also make αβ^+^ effector T cells refractory to Treg cells suppression *in vivo* (8). This clearly indicates that the presence of γδ T cells at the site of tissue inflammation within the CNS indirectly promote the effector functions of αβ^+^ T cells by restraining their *de novo* conversion into Treg cells and inhibiting the suppressive functions of Treg cells in EAE (7, 8) (Figure 1). Similarly, the role of IL-23 in restraining the suppressive functions of Treg is well described in intestinal inflammation, as the frequency of inducible Foxp3^+^ Treg (iTreg) cells increases in the absence of IL-23 (79). However, it is not clear whether appearance of increased frequency of iTreg cells in the absence of IL-23–IL-23R signaling in the intestinal inflammation is due to loss of IL-23R^+^ γδ T cells functions, which are known to suppress the conversion of conventional T cells into Treg cells (8). Moreover, the importance of γδ T cells in mediating the inhibition of suppressive functions of Treg cells was further elucidated in *Tcrd*^−/−^ mice, as these mice develop attenuated EAE with reduced production of IL-17 due to increased frequency of Treg cells (7, 49). Strikingly, anti-CD25 antibody-mediated depletion of Treg cells in *Tcrd*^−/−^ mice enhanced the development of EAE with increased production of IL-17 (8). Altogether, it suggests that γδ T cells are crucial cellular component in promoting inflammation in EAE by restraining the regulatory functions of Treg cells and promoting the functions inflammatory αβ T cells (Figure 2).

## γδ T Cells: Pathogenic or Protective in EAE?

While some models of EAE suggest that γδ T cells are pathogenic, others suggest that they modulate disease; thus, their precise role in pathogenesis is unclear. Both disease-promoting and disease-preventing functions of γδ T cells were documented in EAE. Deficiency of γδ T cells on B10⋅PL background develop a chronic EAE as compared to the development of monophasic acute EAE in the control mice (30). It has been further shown that γδ T cells regulate chronic inflammation by Fas–FasL-mediated killing of CNS-infiltrating inflammatory T cells (30). These studies clearly suggested the protective role of γδ T cells in EAE development.

Although recent literature on γδ T cells in context of IL-17 production implicated the pathogenic role of these cell types in EAE, a number of studies have ascribed the protective role of γδ T cells in EAE (30, 80, 81). A number of factors, such as using different mice strains in combination with either depleting antibodies or genetic manipulation of γδ T cells, might be contributing to these conflicting observations. Treatment of mice with UC7-13D5 anti-γδ antibody accelerates the onset of EAE (80). Similar results were obtained with the usage of UC7-13D5 antibody in other models of autoimmunity. It is partially identified that different subtypes of γδ T cells such as Vγ1 produce regulatory or Vγ4 and Vγ6 produce inflammatory cytokines (Table 1); therefore, it is possible that the treatment of UC7-13D5 antibody may alter this ratio and activate different subtypes of γδ T cell populations by cross-linking their TCR at different phases of EAE, which results in different outcome of disease. Nonetheless, it was not clearly understood whether anti-pan γδ T cells antibody depletes or activates γδ T cells by cross-linking their TCR in EAE (80). Using Tcrd-GFP knock-in mice, it has been clearly demonstrated that treatment with anti-pan γδ T cell antibodies activates, rather than depletes, γδ T cells and therefore exacerbating EAE (34). In addition, *Tcrd*^−/−^ mice develop chronic inflammation in some mouse model of EAE (82). *Tcrd*^−/−^ mice are devoid of δ TCR, which allow γδ T cells not to be activated by their TCR stimulation; however, the number of γδ T cells in these mice remains unchanged. This indicates that TCR-independent activation of γδ T cells can still occur in *Tcrd*^−/−^ mice. We have discussed those different subsets of γδ T cells play opposite roles in EAE development. An interesting dichotomy has been established among Vγ1 and Vγ4 subsets of γδ T cells in EAE, which further provide a logical explanation for previously published contradictory results. Specific antibody-mediated activation of Vγ4 γδ T cells promote the development of EAE associated with enhanced production of IL-17 (34). On the other hand, specific antibody-mediated activation of Vγ1 γδ T cells suppressed EAE development (34). Interestingly, it has recently shown that γδ T cells can be activated with proinflammatory cytokines without the requirement of their TCR signals. To further identify the pathogenic or protective role of γδ T cells in EAE, a detailed study, including the involvement of various subtypes of γδ T cells, is required with more definitive tools. Nonetheless, accumulated literature in other autoimmunity has suggested that γδ T cells might play a pathogenic role in EAE. We have summarized the chief findings of γδ T cells in EAE in Table 2.

**Table 1:** **Major γδ T cells subset in mouse**.

γ/δ usage	Characteristic	Tissue location
Vγ1	Produce IL-4. Regulatory functions in EAE by promoting Treg cells functions (34)	Majorly found in circulation, lymphatics, spleen, lymph nodes
Vγ4	Produce IL-17 and express IL-23R. Promote EAE and CIA. Also promote virus-induced encephalitis (6–8, 27, 35, 83)	Lymphoid tissue and lung, also found in CNS in EAE
Vγ5	Regulation of skin inflammation by maintaining the epidermal homeostasis (84, 85)	Skin and epidermis
Vγ6	Produce IL-17, IL-22, IFN-γ, and express IL-23R (27)	Mucosal tissues, reproductive tract, tongue, lung and kidney. Also detected in CNS during EAE
Vγ7	Prevent colitis by protecting intestinal barrier functions (57, 86, 87)	IEL and intestine

**Table 2:** **Chief findings of γδ T cells in EAE**.

Gene deficiency/treatment	Consequence	Effect in EAE
Anti-γδ T cells (clone GL3) monoclonal antibody treatment in EAE	Reduction in disease pathology. Significant reduction in clinical sign in acute phase of EAE	Protection (52)
Anti-γδ T cells (clone UC7-13D5) monoclonal antibody treatment in EAE	Significant reduction in demyelination and reduction in limb paresis	Protection (88)
Active EAE development in delta (d) chain-deficient mice	Significant reduction in clinical score of EAE with enhanced frequency of Foxp3^+^ Tregs	Protection (8, 49)
EAE induction by adoptively transferring MOG-specific Wt T cells into delta (d) chain-deficient mice	Significant reduction in clinical score of EAE with no cellular infiltration in CNS	Protection (49)
MBP-specific γδ T cells depleted (clone: GL3) lymph node cells were adoptively transferred to induce EAE	Significant reduction in clinical score in EAE with a significant reduction in IL-12 production	Protection (51)
Activation of Vγ4 subset with anti-Vγ4 TCR (UC3) antibody treatment in EAE	Worsen EAE with enhanced IL-17 response	Promote EAE (34)
Activation of Vγ1 subset by anti-Vγ1 TCR antibody (2.11) treatment in EAE	Significant reduction in clinical score of EAE with less proinflammatory cytokines production	Protection (34)
EAE in IL-23R-deficient mice and effect of IL-23–IL-23R axis on γδ T cells	IL-23R-deficient mice are resistant to EAE. γδ T cells constitutively express IL-23R. Almost all γδ T cells express IL-23R in CNS in EAE and produce IL-17	Protection (8, 13)
EAE in IL-18R-deficeint mice and effect of IL-18R on γδ T cells	IL-18R-deficient mice are protected from EAE. IL-18R^−/−^ failed to produce IL-17	Protection (9, 67)
EAE in IL-1R-deficent mice and effect of IL-1R on γδ T cells	IL-1R-deficient mice are protected from EAE. IL-1R1^−/−^ γδ T cells are defective in IL-17 and GM-CSF production in EAE	Protection (7, 10, 62)
EAE in caspase-1-deficient mice and effect of caspase-1 on γδ T cells	Significantly reduced clinical sign of EAE. Defective production of IL-17 and GM-CSF from caspase-1-deficient γδ T cells	Protection (9, 10, 89)

## Relevance of γδ T Cells in Multiple Sclerosis

Multiple sclerosis is demyelinating disease of CNS, which is caused by inflammatory T cells. In addition to αβ^+^ CD4^+^ T cells, γδ T cells were also clearly implicated in the disease pathogenesis in MS. It is shown that γδ T cells are accumulated in the MS plaques (90, 91). A restricted repertoire of γδ T cells was identified in MS lesions. CNS-restricted γδ T cells abundantly express variable gene segments Vδ1 and Vδ2. Furthermore, Vγ9^+^ γδ T cells circulate abundantly in the blood of MS patients and can be used as an indicator of disease activity (92). With the emerging literature on γδ T cells in EAE, it is indicated the involvement of γδ T cells in the pathogenesis of disease (see Table 1). Mouse data in EAE clearly indicated that IL-17-producing γδ T cells are crucial for disease induction and tissue inflammation in EAE (8, 10). Moreover, the role of IL-23, IL-1, IL-18, and caspase-1 is clearly indicated in enhancing IL-17- and GM-CSF-producing γδ T cells in EAE. Recent advancements in understanding the biology of Th17- and IL-17-producing γδ T cell and their implication in autoimmune diseases, including MS, could suggest new therapeutic targets for MS by targeting Th17- and IL-17-producing γδ T cells populations.

## Conclusion

A number of studies have demonstrated a potential role of γδ T cells in the induction and maintenance of demyelinating CNS inflammation. γδ T cells are multifaceted cells, which are equipped with variety of functions to potentially influence all levels of inflammation by recognizing diverse array of antigens, rapid production of inflammatory mediators, and influencing the differentiation of their αβ counterparts. Equipped with functions of both innate and adaptive immune cells, γδ T cells can provide consequential functions in EAE development. Opposing roles of different subtypes of γδ T cells have been described in different mouse strains in EAE. Moreover, the identification of IL-17-producing inflammatory γδ T cells suggested their pathogenic role in EAE. In fact, many of the key questions in autoimmune inflammation, including EAE, were resolved by the discovery of IL-17-secreting Th17 cells. Moreover, clarification on the indispensible role of IL-23–IL-23R axis in Th17 cells also urged researchers to identify the role of IL-23–IL-23R signaling in γδ T cells as they have high expression of IL-23R receptor and therefore are responsive to IL-23 even in steady state – a characteristic which naive αβ T cells lack. This revisits the importance of IL-23 in the settings of EAE since it can influence the generation of two pathogenic subsets Th17 cells and γδT17 cells both of which contributes IL-17 to large extent. Synergistic action of IL-23, IL-1β, and IL-21 induces inflammatory IL-17-producing-γδ T cells, which not only enhance the generation and functions of αβ^+^ Th17 cells but also obstructs the suppressive functions of Treg cells in EAE. Recently, significant progress has been made in understanding the pathogenic role of γδ T cells in tissue inflammation. Yet more substantial evidences are required on different subtypes of γδ T cells for defining their opposing roles in tissue inflammation and explaining the confounding findings on their pathogenic or protective role in EAE.

## Conflict of Interest Statement

The authors declare that the research was conducted in the absence of any commercial or financial relationships that could be construed as a potential conflict of interest.
